# Laparoscopic vs. open procedure for intermediate‑ and high‑risk endometrial cancer: a minimum 4-year follow-up analysis

**DOI:** 10.1186/s12885-022-10301-3

**Published:** 2022-11-23

**Authors:** Xing Zhou, Sitian Wei, Qingchun Shao, Jun Zhang, Rong Zhao, Rui Shi, Wei Zhang, Kejun Dong, Wan Shu, Hongbo Wang

**Affiliations:** Department of Obstetrics and Gynecology, Union Hospital, Tongji Medical College, Huazhong University of Science and Technology, Wuhan, Hubei China

**Keywords:** Endometrial cancer, Intermediate‑ and high‑risk, Laparoscopic surgery, Open procedure, Postoperative complication, Outcomes

## Abstract

**Background:**

The long-term oncologic outcomes after laparoscopic and open procedures for patients with intermediate‑ and high‑risk endometrial cancer (EC) remain unclear. Accordingly, laparoscopy cannot still be recommended as the standard choice for intermediate‑ and high‑risk EC. This retrospective study aimed to assess the perioperative and long-term oncologic outcomes of laparoscopy and open surgery in patients with intermediate- and high‑risk ECs within a minimum 4-year follow-up.

**Methods:**

We included 201 patients who underwent laparoscopic or open procedures for intermediate‑ and high‑risk EC between 2010 and 2017. Between-procedure comparisons of perioperative and oncological outcomes were performed using the independent t-test or Pearson’s chi-squared test and the Kaplan–Meier method, respectively.

**Results:**

Finally, there were 136 intermediate‑ and 65 high‑risk endometrial tumors in the laparoscopic and open groups, respectively. There were no between-group differences in all baseline characteristics. Compared with the open group, the laparoscopic group had a significantly longer mean operating time (*p* = 0.005) and a lower mean estimated blood loss (EBL) (*p* = 0.031). There was a higher possibility of postoperative complication in the open group than in the laparoscopic group (*p* = 0.048). There were no significant between-group differences in pathological outcomes as well as the recurrence-free survival and overall survival rates (*p* = 0.626 and *p* = 0.148, respectively).

**Conclusions:**

Among patients with intermediate‑ and high‑risk EC, laparoscopic surgery has an advantage over the open surgery in reducing EBL and the rate of postoperative complications without weakening the oncological control. There were no between-procedure differences in the recurrence-free and overall survival rates.

## Introduction

Worldwide, endometrial cancer (EC) is the 6^th^ most commonly diagnosed cancer [1] and the 14^th^ leading cause of cancer-related death among women [2]. EC can be divided into several histopathological subtypes with differences in tumor biology, which leads to different clinical outcomes [3]. Based on the pathological characteristics, EC can be classified as a low-, intermediate-, or high-risk disease [4]. According to the Japan Society of Gynecologic Oncology guidelines for treating uterine body neoplasms published in 2013, the classifications can be described as follows [5]: (1) low-risk (grade 1 or 2 tumors with < 1/2 myometrial invasion), (2) intermediate-risk (grade 1 or 2 tumors with ≥ 1/2 myometrial invasion, or grade 3 tumor with < 1/2 myometrial invasion, and lymphovascular space invasion), and (3) high-risk disease (grade 3 tumors with ≥ 1/2 myometrial invasion and cervical stromal invasion).

For most women with EC, the current treatment modalities include the removal of the uterus, cervix, fallopian tubes, and ovaries as well as sentinel lymph node evaluation [6]. Traditionally, open surgery has been used to treat EC. However, laparoscopic surgery has become increasingly popular given its advantages in reducing surgical morbidity and accelerating postoperative recovery compared with open surgery [7, 8]. Laparoscopic surgery for EC treatment has become common over the last two decades [9]. However, compared with open surgery, minimally invasive surgery (MIS) has been shown to have a higher recurrence risk in patients with intermediate- and high-risk EC [10–13]. This could be attributed to tumor manipulation during endoscopic surgery or the specific conditions of endoscopy. Moreover, cancer control using laparoscopic and open surgery remains unclear with respect to oncological long-term outcomes, such as recurrence-free survival (RFS) and overall survival (OS) [8].

Considering the conflicts of the above study results, in our institution, researchers collected the clinical information of patients who were diagnosed with endometrial cancer and underwent surgery, and followed up for at least four years, since 2010. This study aimed to compare the perioperative and long-term oncologic outcomes between laparoscopic and open surgery in patients with intermediate- and high-risk EC during a minimum 4-year follow-up period.

## Materials and methods

### Study population

In this single-center retrospective cohort study, we analyzed baseline demographics and clinical characteristics obtained from our prospectively maintained database at Wuhan Union Hospital, China. Among 679 female patients, we included 201 patients between January 2010 and December 2016 based on the following inclusion criteria: (a) patients with intermediate- or high-risk ECs; (b) a minimum 4-year follow-up; (c) having undergone laparoscopic or open surgery for ECs; and (d) having complete clinical/histological data and follow-up outcomes. Patients eligible for all these criteria at the same time were then included in the study after signing an informed consent form. 201 patients were divided into the laparoscopic (*n* = 136) and open (*n* = 65) groups, based on the surgical type. All the patients underwent routine preoperative computed tomography (CT) to estimate the presence of distant metastases. All procedures were performed by surgeons with extensive training and experience in gynecologic oncology and advanced laparoscopic surgery. The surgical approach was selected at the discretion of highly experienced surgeons based on the tumor and patient characteristics. The existing studies presented the detailed procedures of laparoscopic and open methods for EC [14–16]. Pelvic lymphadenectomy was performed in patients with a tumor diameter > 2 cm, while pelvic-para-aortic lymphadenectomy was performed in patients with grade 3 tumors, more than 50% myometrial invasion, type 2 histopathology, and higher stages than stage 1A.

### Data selection

Baseline demographics and clinical features (age, diabetes mellitus, hypertension, delivery times, surgical extension, and adjuvant therapy) were extracted from the database. Additionally, we analyzed perioperative outcomes, including surgical approach, operating time (OT), estimated blood loss (EBL), transfusion, and postoperative complications graded following the Clavien-Dindo classification [17]. Pathological outcomes included pathological stage and grade, histological subtype, and lymph node metastasis. Postoperative follow-up was performed at regular intervals for each patient. Overall survival (OS) and recurrence-free survival (RFS) were defined as the interval from the surgery date to any-cause mortality and recurrence or metastasis, respectively.

### Statistical analysis

Statistical analyses were performed using SPSS (version 25.0) and Excel (version 2004). All continuous variables were presented as mean and standard deviation (mean ± SD) and were analyzed using the independent t-test. Categorical variables were compared using Pearson’s chi-squared or Fisher’s exact test. Survival outcomes of patients with malignant tumors were evaluated using the Kaplan–Meier method with a log-rank test. Statistical significance was set at a two-tailed *P* value of 0.05.

## Results

### All study patients

**Fig. 1:**
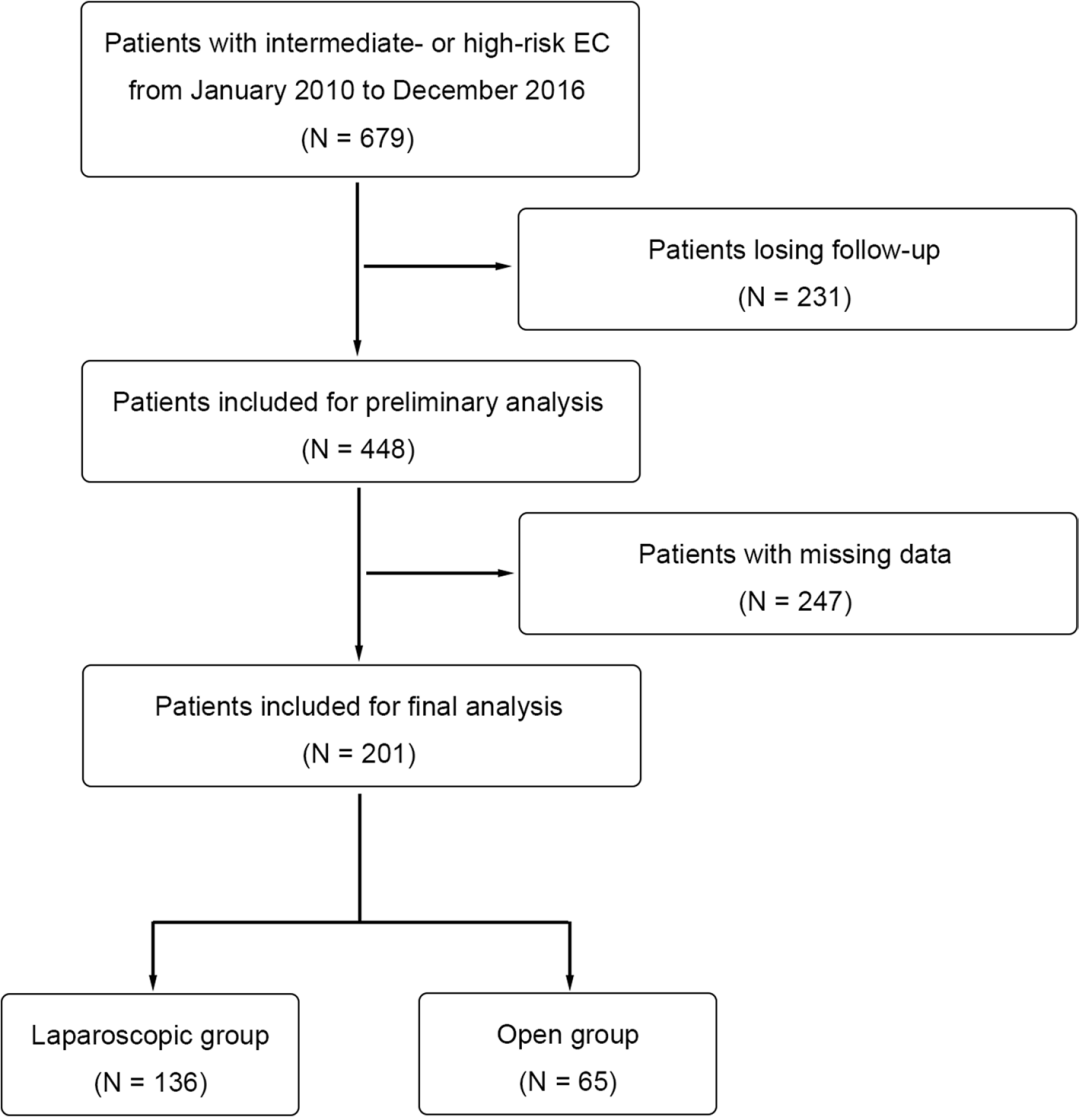
Consort diagram for patients selection

In total, 201 patients were included in the analysis (Fig. 1). Among them, 136 and 65 patients were in the laparoscopic and open groups, respectively. All the preoperative characteristics had been presented in detail in Table 1. The mean age of patients in the laparoscopic and open groups at initial diagnosis was 53.1 ± 7.9 and 52.9 ± 10.4 years, respectively. In the laparoscopic group, the mean body-mass index (BMI) was 24.44 ± 3.60 kg/m^2^, the median number of partus was 2, 21.3% (29/136) had hypertension, and 12.5% (17/136) had diabetes. In the open group, the BMI was 23.99 ± 3.59 kg/m^2^, the median number of partus was 2, 24.6% (16/65) had hypertension, and 13.8% (9/65) had diabetes. There were no significant between-group differences in the aforementioned demographic characteristics.

**Table 1:** Demographic data and medical history of patients in the laparoscopic and open groups

	Laparoscopic approach (*n* = 136)	Open approach (*n* = 65)	*p* value
Age, years, mean ± SD	53.1 ± 7.9	52.9 ± 10.4	0.906
BMI, kg/m^2^, mean ± SD	24.44 ± 3.60	23.99 ± 3.59	0.404
Partus, n, median (Range)	2 (1, 2)	2 (1, 2.5)	0.545
Hypertension (Yes), n (%)	29 (21.3%)	16 (24.6%)	0.6
Diabetes (Yes), n (%)	17 (12.5%)	9 (13.8%)	0.79
Surgical extension, n (%)			0.35
LH + BSO + pelvic LND	51 (37.5%)	20 (30.8%)	
LH + BSO + pelvic and para-aortic LND	85 (62.5%)	45 (69.2%)	
Adjuvant therapy, n (%)			0.329
No adjuvant therapy	79 (58.1%)	42 (64.6%)	
Chemotherapy	39 (28.7%)	12 (18.5%)	
External radiation	14 (10.3%)	10 (15.4%)	
Chemotherapy + external radiation	4 (2.9%)	1 (1.5%)	

*SD* Standard deviation, *BMI* Body-mass index, *LH* Hysterectomy, *BSO* Bilateral salpingo-oophorectomy, *LND* lymphadenectomy

Additionally, there were no statistically significant between-group differences in the therapeutic regimens. The therapeutic regimen combining hysterectomy, bilateral salpingo-oophorectomy, and lymphadenectomy (LND) was performed on each patient in both groups. Further, 85 (62.5%) and 45 (69.2%) patients underwent para-aortic LND in the laparoscopic and open groups, respectively (*p* = 0.350). Moreover, 79 (58.1%) and 42 (64.6%) patients, in the laparoscopic and open groups, respectively, did not receive any adjuvant therapies, including chemotherapy and external radiation (*p* = 0.329).

### Comparisons by intraoperative conditions and histopathological outcomes

Next, we performed between-group comparisons of the surgical conditions and histopathological outcomes. Results had been exhibited in Table 2. Compared with the open group, the laparoscopic group had a significantly longer mean operative time (OT) (3.7 vs. 3.3 h, *p* = 0.005) and lower mean estimated blood loss (EBL) (190.90 vs. 247.87 ml, *p* = 0.031). There were no significant between-group differences in the rates of all pathological outcomes, including pathologic stage, pathologic grade, and histologic subtype (*p* = 0.372, *p* = 0.791, and *p* = 0.816, respectively). Moreover, there was no significant difference in the median lymph node count between the laparoscopic and open groups (30 vs. 29, *p* = 0.959). 8 (5.9%) and 2 (3.1%) cases had positive lymph nodes in the laparoscopic and open groups, respectively (*p* = 0.505).

**Table 2:** Intraoperative and histopathological outcomes and postoperative adjuvant therapy

	Laparoscopic approach (*n* = 136)	Open approach (*n* = 65)	*p* value
Operative time, hours, mean ± SD	3.7 ± 1.3	3.3 ± 1.0	0.005
Estimated blood loss, ml, mean ± SD	190.90 ± 133.43	247.87 ± 201.71	0.031
Lymph node count, median (Range)	30 (19.5, 37)	29 (14.5, 38)	0.959
Lymph node metastases, n (%)	8 (5.9%)	2 (3.1%)	0.505
Stage, n (%)			0.372
I	119 (87.5%)	61 (93.8%)	
II	12 (8.8%)	2 (3.1%)	
III	5 (3.7%)	2 (3.1%)	
Grade, n (%)			0.791
G1	69 (50.7%)	31 (47.7%)	
G2	43 (31.6%)	24 (36.9%)	
G3	24 (17.6%)	10 (15.4%)	
Postoperative histology, n (%)			0.816
Endometrioid adenocarcinoma FIGO grade I-II	77 (56.6%)	38 (58.5%)	
Endometrioid adenocarcinoma FIGO grade III	48 (35.3%)	24 (36.9%)	
Serous adenocarcinoma	5 (3.7%)	1 (1.5%)	
Clear cell adenocarcinoma	3 (2.2%)	2 (3.1%)	
Carcinosarcoma	3 (2.2%)	0 (0.0%)	

### Perioperative complications and follow-up outcomes for two groups

**Fig. 2:**
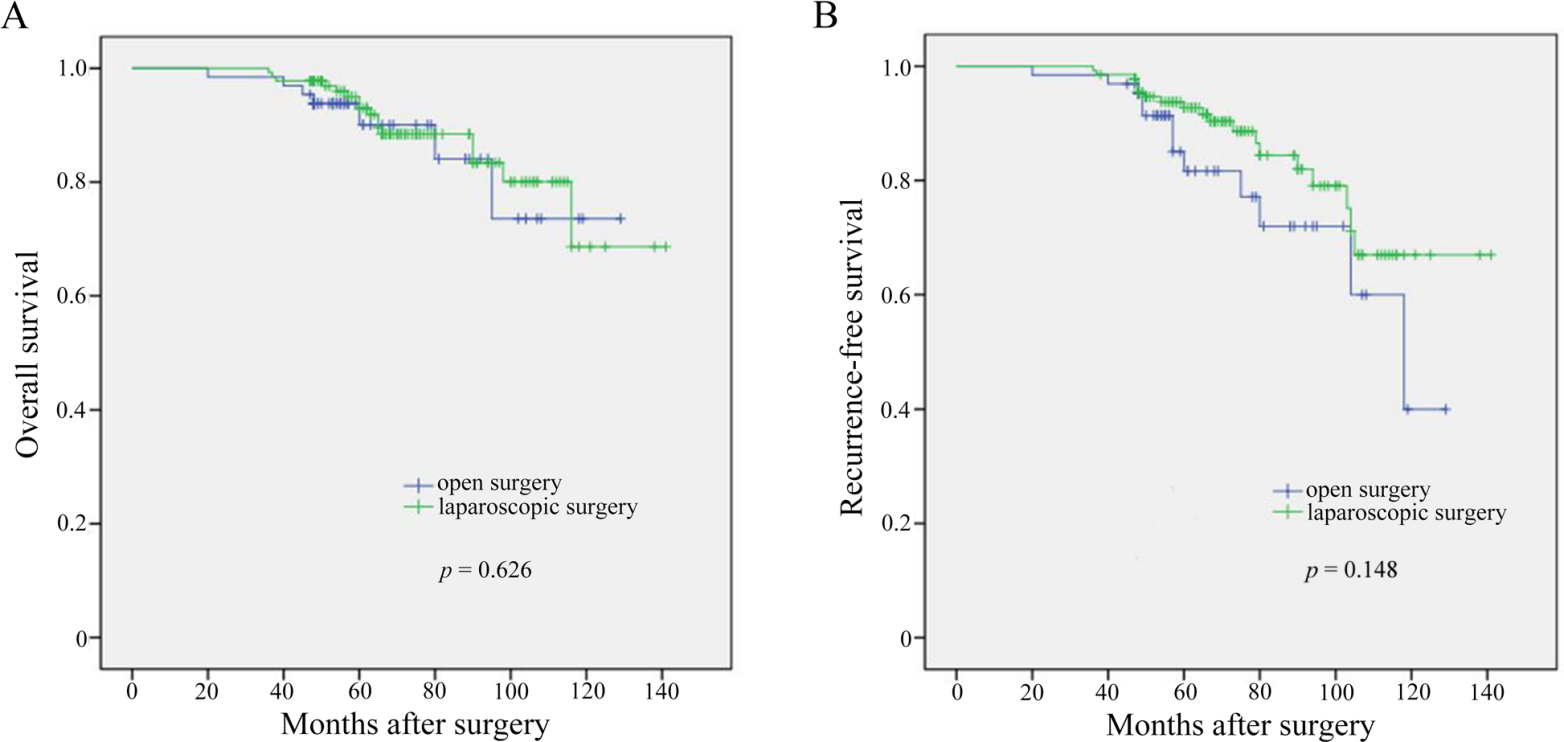
Kaplan–Meier survival curves for overall survival (**A**) and recurrence-free survival (**B**)

As shown in Table 3, the open group had a significantly higher proportion of postoperative complications than the laparoscopic group (*p* = 0.048). Specifically, 18.5% (12/65) and 7.4% (10/136) of patients in the open and laparoscopic groups, respectively, showed complications of varying degrees, including hematuria, thrombosis, and intestinal obstruction. After discharge, the median (interquartile range) follow-up duration in the laparoscopic and open groups was 67.5 (55.3–90) and 56 (48–78.5) months, respectively. During the follow-up intervals, overall-death events occurred in 7 and 10 patients in the laparoscopic and open groups, respectively, while recurrence events occurred in 10 and 17 patients in the laparoscopic and open groups, respectively. Kaplan–Meier analysis revealed no significant between-group differences in OS and RFS (Fig. 2).

**Table 3:** The perioperative complications and follow-up outcomes for two groups

	Laparoscopic approach (*n* = 136)	Open approach (n *n* = 65)	*p* value
Postoperative complication, n (%)			0.048
low grade (Clavien–Dindo I–II)	8 (5.9%)	10 (15.4%)	
high grade (Clavien–Dindo III–IV)	2 (1.5%)	2 (3.1%)	
Follow-up time, months, median (Range)	67.5 (55.3–90)	56 (48–78.5)	0.501
Disease recurrence, n (%)	10 (7.4%)	17 (26.2%)	< 0.001
Death events, n (%)	7 (5.1%)	10 (15.4%)	0.017

### Comparison of clinical characteristics of EC patients undergoing laparoscopic and open surgery in different institutions

Then, we compared the clinical characteristics of EC patients undergoing laparoscopic and open surgery in our and other 4 institutions (Table 4). Baseline characteristics such as age distribution did not differ between the two groups. Consistent with our results, in most institutions, the operation time of the laparoscopy group was longer than that of the open group, but EBL was relatively less. The opposite situation also happened, which might be caused by different surgical methods and conditions in different hospitals. Different centers had distinct rates of disease recurrence and death events. The disease recurrence rate ranged from 4.6–31.7%, while the occurrence rate of death events was 3.9–24.6%. These results indicated that different surgical methods might bring different prognoses in patients with ECs in different institutions.

**Table 4:** Comparison of the clinical characteristics of EC patients undergoing laparoscopic and open surgery in different institutions

Author (Year)	Surgery type	Age, years	OT, hours	EBL, ml	Disease recurrence, n (%)	Death events, n (%)
Present study	Laparoscopic approach (*n* = 136)	53.1 ± 7.9	3.7 ± 1.3	190.90 ± 133.43	10 (7.4%)	7 (5.1%)
	Open approach (*n* = 65)	52.9 ± 10.4	3.3 ± 1.0	247.87 ± 201.71	17 (26.2%)	10 (15.4%)
Monterossi (2017) [18]	Laparoscopic approach (*n* = 141)	67 (40–92)	2.7 (0.7–8.0)	100 (10–500)	**25 (17.7%)**	19 (13.5%)
	Open approach (*n* = 142)	69 (37–90)	2.6 (0.8–7.4)	250 (20–1000)	**45 (31.7%)**	35 (24.6%)
Zhang (2013) [19]	Laparoscopic approach (*n* = 151)	56.6 (27–82)	3.5 (1.7–7.7)	86 (5–450)	7 (4.6%)	9 (6.0%)
	Open approach (*n* = 121)	57.2 (29–79)	3.9 (1.5–9.5)	419 (20–4000)	6 (5.0%)	12 (9.9%)
Janda (2017) [20]	Laparoscopic approach (*n* = 407)	63.3 ± 10.0	2.2 (0.8–5.0)	unknown	33 (8.1%)	16 (3.9%)
	Open approach (*n* = 353)	63.1 ± 10.6	1.8 (0.6–4.2)	unknown	28 (7.9%)	14 (4.0%)
Walker (2012) [21]	Laparoscopic approach (*n* = 1696)	62.8 (55.4–71.6)	unknown	unknown	210 (12.4%)	229 (13.5%)
	Open approach (*n* = 920)	62.7 (54.9–70.6)	unknown	unknown	99 (10.8%)	121 (13.2%)

## Discussion

Endometrial cancer is the second most common gynecologic malignancy worldwide; further, its main treatment modalities are total hysterectomy and bilateral salpingo-oophorectomy. Minimally invasive surgery (MIS) is the preferred surgical approach for patients with EC given the reduced hospital stay, decreased blood loss, and mild discomfort. Several studies have reported the oncological outcomes of laparoscopic and open surgeries for EC [22–28]; however, most of the studies concentrated on patients with low-risk EC and short-term follow-up. Further, there were extremely limited reliable studies concerning oncological long-term outcomes of laparoscopic and open surgeries in the literature. Fabio et al. reported that patients with ECs who underwent laparoscopy and laparotomy had similar recurrence-free survival rates and overall survival rates during a minimum 3-year follow-up [29]. However, several studies had contradicted the assumption that MIS was more beneficial to patients with EC, with reports that MIS involved a higher risk of recurrence than open surgery. Accordingly, future studies are warranted to determine whether the laparoscopic approach is the preferable choice for intermediate- and high-risk ECs.

In our study cohort, we included 201 patients with intermediate- or high-risk EC, of whom 136 underwent laparoscopic surgery and 65 underwent open surgery. There were no significant between-group differences in the baseline clinical information, including age, BMI, number of partus, and presence of hypertension or diabetes, as well as the treatment strategies. Regarding the intraoperative situation, the laparoscopic group had a longer operation time than the open group, which was consistent with previous reports [30, 31]. However, the laparoscopic group had a reduced amount of intraoperative bleeding, which could be attributed to a smaller wound, limited field of vision, and minimal invasiveness of the operation. Postoperative complications might occur in both laparoscopic and open surgery; however, the laparoscopic group had a lower probability of complications. Additionally, compared with the open group, the laparoscopic groups showed lower mortality and recurrence rates; however, there were no significant between-group differences in OS and RFS.

MIS was introduced for cancer management to decrease the mortality rate while obtaining similar oncologic outcomes as open surgery. However, it remains unclear whether laparoscopy is better than open surgery in EC or other tumors such as kidney cancer [32] and cervical cancer [33]. Nonetheless, it has been determined that although laparoscopic surgery prolongs the operation time, it reduces intraoperative bleeding and postoperative complications. Manipulation under an endoscopic camera is inevitably more difficult than under direct vision, especially when a high number of lymph nodes must be intraoperative removed. However, although laparoscopic surgery has shown incidences of postoperative complications ranging from 1.6 to 29.9% in published studies [34–39], the laparoscopic group had a lower proportion of postoperative complications than the open group given the small incision and minimized injury.

Since Fader et al. reported the first study on laparoscopic management of high-grade and type 2 endometrial carcinomas (*n* = 191) in a multicenter retrospective study in 2012 and concluded that high-risk EC was not a contraindication for MIS [36], MIS has been widely used for various graded ECs. The results of our cohort data described that the two approaches yielded similar overall survival and recurrence-free survival, which was consistent with previous reports by Favero et al. [39] and Koskas et al. [38]. This indicates that laparoscopic surgery does not reduce oncological safety or efficacy compared with open surgery. There have been concerns that circulating CO_2_ could cause spillage of cancer cells in the peritoneal cavity [40]. However, we observed no significant between-group differences in the recurrence and any-cause death rates, indicating that the insufflation gas did not cause tumor spillage in our cohort.

Our study has several limitations. First, since this was a retrospective study, there are weaknesses with respect to the extraction of baseline characteristics and follow-up data. Accordingly, certain complications might have been underestimated, especially low-grade postoperative complications, even with the thorough scrutiny of medical records. Secondly, this was a single-center study; moreover, the surgical methods and operations were easily affected by the habits of the doctors. Therefore, multicenter studies are warranted. Third, this study had a short follow-up time; moreover, the dimensions were narrow, which did not allow a comprehensive and objective evaluation of the advantages and disadvantages of each surgery type.

## Conclusion

Among patients with intermediate‑ and high‑risk EC, laparoscopic surgery has an advantage over open surgery with respect to reducing EBL and the rate of postoperative complications without compromising the OS and RFS. Our findings could provide guidance and suggestions for patients with EC and doctors when choosing treatment approaches for EC.

## Data Availability

The datasets used and/or analysed during the current study available from the corresponding author on reasonable request.

## Abbreviations

EC: Endometrial cancer
EBL: Estimated blood loss
RFS: Recurrence-free survival
OS: Overall survival
CT: Computed tomography
OT: Operating time
BMI: Body-mass index
LND: Lymphadenectomy
SD: Standard deviation
LH: Hysterectomy
BSO: Bilateral salpingo-oophorectomy
MIS: Minimally invasive surgery
